# Blunt apical dissection during anatomic radical retropubic prostatectomy

**DOI:** 10.1186/1756-0500-2-20

**Published:** 2009-02-06

**Authors:** Kazunori Namiki, Ali Kasraeian, Saif Yacoub, Charles J Rosser

**Affiliations:** 1 Department of Urology, University of Florida, Gainesville, FL, USA

## Abstract

**Background:**

Meticulous apical dissection during a radical prostatectomy is imperative to achieve desirable pathologic and quality of life outcomes.

**Findings:**

We describe a novel technique using careful blunt dissection to better delineate the apex of the prostate, providing a simple means to potentially lessen positive surgical margins at the apex and promote better continence and erectile function in men undergoing an anatomic radical prostatectomy.

Median operative time and blood loss were 190 minutes and 675 mL, respectively. Only 10 percent of the patients with positive surgical margins were found to have apical positive surgical margins. Ninety-three percent of patients reported no urinary leakage.

**Conclusion:**

We believe our technique of isolating the DVC with blunt dissection and then ligating and transecting the DVC to be feasible approach that requires larger studies to truly confirm its utility.

## Findings

In the US, radical prostatectomy (RP) is the most common treatment for localized prostate cancer [1] and results in durable, disease-free survival with few complications [2,3]. The durable disease-free survival and low complication rates are in part due to the meticulous apical dissection of the prostate which translates into less blood loss and improved visualization of critical structures [4]. Optimal visualization leads to reduction in positive apical surgical margin rates as well as improvement in the dissection of the urethra and caveronosal nerves which are critical when addressing post-prostatectomy continence and erectile dysfunction, respectively. This concept has been clearly illustrated previously by Walsh and Donker, who reported using sharp dissection to create a plane between the dorsal venous complex (DVC) and urethra [4,5]. Inappropriate sharp dissection can cause bleeding and may inadvertently injure the rhabdosphincter. A natural plane exists between the DVC and urethra that can be identified through careful blunt dissection. Herein, we report an effective method to optimally dissect the apex of the prostate and to assist in identifying and ligating the DVC.

### Key Surgical Technique Steps

**Figure 1:**
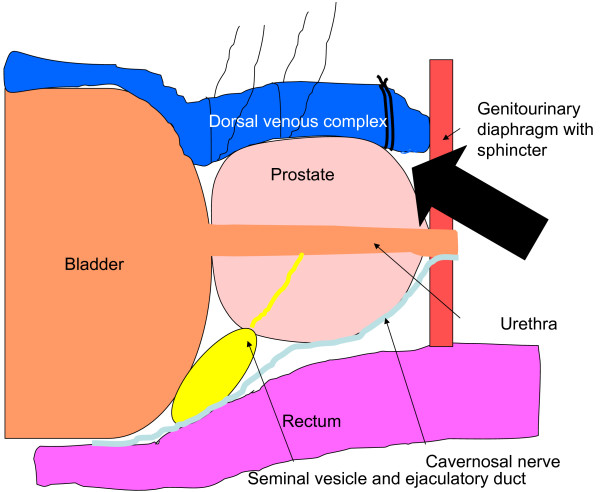
**After the endopelvic fascia has been incised bilaterally, blunt dissection is used to create a groove between the urethra and dorsal venous complex (DVC)**. A Mixter forceps is then passed in this groove and a #1 Vicryl tie is used to ligate the DVC. This maneuver helps to clearly delineate the apex and DVC. Next, two figure eight Vicryl sutures are placed as proximal as possible on the DVC. Lastly, two figure eight Vicryl suture (one at the bladder neck and another at the mid portion of the prostate on its anterior surface) are placed to minimize back bleeding. Now with the DVC secured and optimal vision of the apex of the prostate, the DVC may be transected.

Patients underwent an anatomic radical retropubic prostatectomy via a 9 cm infraumblical incision. The space of Reituz was developed and a self-retaining retractor was used to expose the pelvis. Intermediate or high risk patients (i.e., PSA ≥ 10 ng/ml, Gleason score ≥ 7, or ≥ clinical stage T3) underwent a standard bilateral pelvic lymph node dissection. Next, the endopelvic fascia was incised bilaterally with electrocautery and the levator muscle fibers were swept off the anterior and lateral surfaces of the prostate. Electrocautery was not used for any other portion of the case in an attempt to prevent injury to the cavernosal nerves. Subsequently, two figure of eight sutures (2-0 Vicryl with a CT-1 needle) were placed at the base and mid portion of the prostate to minimize back bleeding (Figure 1). Puboprostatic ligaments were not transected. Utilizing gentle blunt dissection with the right index finger, a groove was created between the urethral and dorsal venous complex (DVC) (Figure 1), this is different to previous reports where sharp dissection with a McDougal clamp was employed [5]. A Mixter forceps was used to pass a #1 Vicryl tie around the isolated DVC which was subsequently ligated. This tie is used to better identify the DVC and is routinely cut during transection of DVC. Next, two figure of eight sutures (2-0 Vicryl with a CT-2 needle) were utilized to further ligate the most proximal extent of the isolated DVC. A Mixter forceps was passed posterior to the DVC, which was transected with a 15 blade knife. Rarely will further hemostatic sutures be required in the ligated DVC. At this time, the urethra is clearly identified. Tissue lateral to the urethra was dissected freely and released from the urethra with Metzenbaum scissors. A Mixter forceps is passed inside this tissue and posterior to the urethra. An umbilical tape was passed in the right angle clamp, clearly identifying the urethra. The Foley catheter is lubricated at the urethral meatus and then disconnected from its drainage bag. The anterior surface of the urethra is transected. The external portion of the Foley catheter is transected and eventually brought into the pelvic wound. The posterior aspect of the urethra is transected. The urethralis muscle is transected. The remainder of the procedure was performed as previously reported [4,5].

### Outcomes

After IRB approval was obtained, the charts of 54 men with a median age of 64 years (range, 45–74 years) were reviewed. Median follow-up of the cohort was 36 months. A description of the study population, including race, serum PSA, Gleason score of prostate biopsy, and clinical stage, is shown in Table 1. Of the 54 men, 14 (26%) underwent a bilateral nerve sparing RP; 28 (52%) men underwent a unilateral nerve sparing RP; and 12 (22%) men underwent non-nerve sparing procedures. Median operative time and blood loss were 190 minutes and 675 mL, respectively. Organ confined disease (pT2) was diagnosed in 76% of the patients, whereas 31% of patients had poorly differentiated tumors. The overall positive surgical margin rate was 18% (95% CI 0.07–0.27). Only 10 percent of the patients with positive surgical margins were found to have apical positive surgical margins (Table 1). Thirty-five percent of the individuals with positive surgical margins and/or positive lymph node were treated with adjuvant or salvage hormonal therapy or a combination of hormonal therapy and external beam radiation therapy. Analyses of the 12 month EPIC questionnaire responses under urinary function revealed only one subject reported no urinary control whatsoever. This patient had a previous history of proximal urethral stricture treated by incision of stricture. Another six percent of patients reported occasional dribbling, whereas 93% of patients reported total control. No subject less than 65 years reported incontinence. Analyses of the 12 month EPIC questionnaire responses under sexual function revealed 52% of the subjects who were potent prior to surgery rated their ability to function sexually during the last four weeks as good or very good. Of these patients, over 75% were using oral phosphodiesterase type 5 inhibitors with or without a vacuum erection device. In men less than 65 years with no erectile dysfunction prior to surgery, 70% have erections with the use of PDE5 inhibitors alone.

**Table 1:** Characteristics of patient (n = 54) undergoing radical prostatectomy.

	**No. of Patients**	**%**
Age (years)		

Median	64 ± 7 years	

Range	45–74 years	

Race/ethnicity		

White	31	57

Black	21	39

Other	2	4

Clinical tumor classification		

T1c	41	76

T2	11	20

T3	2	4

Preoperative PSA level		

Median (ng/mL)	5.45	

Range (ng/mL)	0.71–24.40	

≤ 4.0	12	22

4.1–10	32	59

> 10	10	19

Biopsy Gleason score		

≤ 6	35	65

3+4	9	17

4+3	4	7

≥ 8	6	11

Prostatectomy Gleason Score		

≤ 6	21	39

3+4	15	28

4+3	1	2

≥ 8	17	31

Pathologic tumor classification		

pT2	41	76

pT3a	10	19

pT3b	2	4

N1 (lymph node pos.)	1	2

Pos. surgical margin	10	18

Apex	1	10

Other	9	90

### Comments

Radical retropubic prostatectomy is a challenging surgical procedure with a known, significant learning curve to achieve optimal outcomes. The ultimate effect of a careful dissection of the apex of the prostate is gauged by measuring surgical outcomes, specifically pathologic and quality-of-life outcomes. We believe that the surgical modifications described in this report should result in a lower incidence of positive apical surgical margin rate, even in patients with clinical Stage T2 disease. Several recent publications have also described modifications to reduce the incidence of positive apical surgical margins. Despite these modifications, apical positive margin rates could not be reduced to below 15% [6-8].

In this study, patients had pathologic outcomes – specifically positive surgical margin rates – that were comparable to those reported in the literature [8-10]. Although it is possible that our improved surgical margin rate was in part related to better patient selection and increased surgical experience, we do not believe that these factors alone account for the very low positive apical surgical margin rate in this study compared with those in other recent series. In fact, we believe the reduced apical surgical margin rates were due to the optimal visualization this technique provided.

The ultimate goal of radical retropubic prostatectomy is cancer control with little to no morbidity. We do not believe our established technique is associated with increased morbidity, seeing that in our patients less than 65 years reported excellent urinary continence rates and favorable potency rates as assessed by their 12 month EPIC questionnaire. Previously we have reported on the outcomes of subjects treated utilizing this technique [11,12]. However, the outcomes from this reported technique should be corroborated by other surgeons.

Radical retropubic prostatectomy continues to be a challenging procedure. Careful apical dissection is needed for optimal results. We believe our technique of isolating the DVC with blunt dissection to be a feasible approach that requires larger studies to truly confirm these encouraging preliminary results.

## Abbreviations

DVC: dorsal venous complex; RP: radical prostatectomy; PSA: prostate specific antigen; EPIC: Expanded Prostate Cancer Index Composite.

## Competing interests

The authors declare that they have no competing interests.

## Authors' contributions

KN analyzed data and drafted the manuscript. AK and SY reviewed patients' charts and collected data. CJR conceived the study, and participated in its design and coordination. All authors read and approved the final manuscript.
